# Cruising and jumping: the effect of microplastics on the swimming behavior of copepods measured by 3D Lagrangian particle tracking velocimetry

**DOI:** 10.1140/epje/s10189-026-00587-7

**Published:** 2026-05-27

**Authors:** F.-G. Michalec, O. Praud, M. Lorite-Diez, S. Cazin, S. Souissi, E. Climent

**Affiliations:** 1 IMFT, Institut de Mécanique des Fluides de Toulouse - UMR 5502 CNRS, Toulouse INP, Univ. Toulouse, Allée Camille Soula, 31400 Toulouse, France; 2 LOG, Laboratoire d’Océanologie et de Géosciences, UMR 8187 CNRS, Univ. Lille, Univ. Littoral Côte d’Opale, 28, avenue Foch, 62930 Wimereux, France; 3 IISTA, Andalusian Institute for Earth System Research, Universities of Granada, Jaén and Córdoba, Av. del Mediterráneo s/n, 18006 Granada, Spain; 4 OCEAN, Operation Center for Enterprise Academia Networking, National Taiwan Ocean University, Keelung, 20224 Taiwan

## Abstract

**Abstract:**

Calanoid copepods are key components of marine and estuarine food webs. Exposure to various classes of pollutants induces changes in their swimming behavior. This raises concerns about potential effects on critical processes such as feeding, mating, predator avoidance and vertical migration. The effect of pollution by microplastics is not well known. We investigated in a large experimental tank the effects of the smallest size fraction of microplastics on the swimming behavior of the estuarine copepod *Eurytemora affinis*. Because the motion of zooplankton is intrinsically linked to that of the ambient fluid, we recorded copepods moving freely in calm water and in grid-generated turbulence to recreate some of the hydrodynamic conditions they experience in their natural environment. Using an advanced implementation of 3D Lagrangian particle tracking velocimetry, we simultaneously measured copepod trajectories and the surrounding flow field at high temporal resolution. In calm water, copepods alternated between periods of cruising and sudden relocation jumps. In turbulence, copepod motion was dominated by transport by the flow, yet jumps allowed copepods to deviate from the flow streamlines. The measurement of the relative velocity of copepods with respect to the underlying flow enabled us to characterize the statistics of these jumps. Turbulence significantly increased jump frequency without modifying their amplitude or duration. Following a 12-hour exposure to polyethylene fragments at 300 $\mu$g/L, copepods showed increased jump frequency in calm water corresponding to 40 % increase in energetic cost. In contrast, exposure to microplastics produced weak additional effects on swimming behavior under turbulent conditions. These results confirm the existence of an active response to turbulence in *E. affinis* and are consistent with a hyperactive behavior triggered by exposure to microplastic pollution.

**Graphic Abstract):**

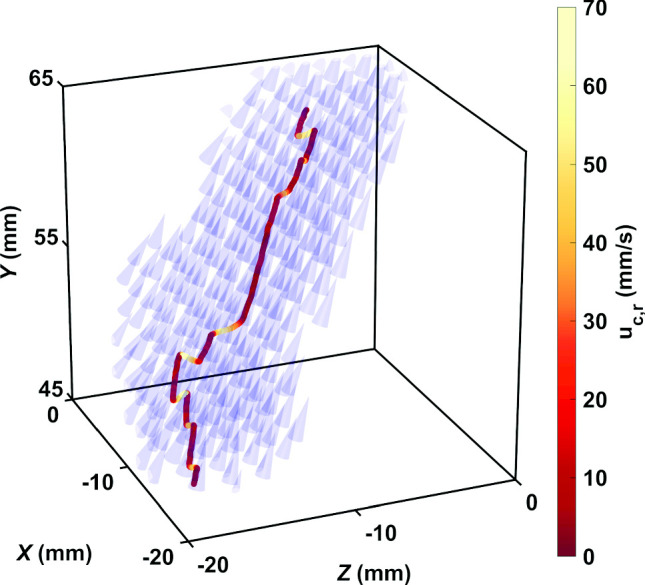

## Introduction

Plankton have been frequently described as organisms that are carried along by the flow because they are unable to swim sufficiently strongly to move against currents. However, many species do not behave as passive particles and are motile, propelling themselves through water at velocities up to several hundreds millimeters per second [1]. They have evolved a variety of behaviors that confer more efficient exploitation of their heterogeneous and fluctuating environment. [2] provided a comprehensive synthesis of the dynamics of motile micro-organisms in turbulent flows, with particular emphasis on swimmer-turbulence coupling, preferential sampling, and behavioral modulation by fluid velocity gradients. A striking illustration is the vertical migration that certain species of phytoplankton and zooplankton perform across the mixed layer of the oceans [3–5].

Calanoid copepods represent an important part of the zooplankton in marine and estuarine ecosystems. They play important roles in the transfer of matter and energy from the primary producers to upper trophic levels [6], they support the development of many larger aquatic organisms including commercially important fishes [7], and they contribute to carbon export in some parts of the ocean [8, 9]. Calanoid copepods swim vigorously with respect to their small size and engage in complex behaviors that are important for their survival and development. Motility allows copepods to swim against upwelling currents to maintain their vertical position in coastal areas [10], to swim away from unfavorable regions of high turbulence at the surface of the ocean [11], to locate and exploit phytoplankton patches [12, 13], to escape from predators [14], and to mate [15]. Consequently, the motility of copepods shapes much of their development at the individual and population levels [16]. Exposure to various types of pollutants affects their swimming behavior [17, 18], which raises concern about their ability to perform active processes that are important for their fitness.

Microplastic fragments are accumulating in the environment on an unprecedented scale. Their toxicity to zooplankton is complex and not well known. Some studies have shown that certain species, from copepods to krill, ingest plastic fragments because they fall within the size range of their phytoplankton diet [19]. The shape of the fragments appears to play an important role in determining their ingestion rate [20, 21]. Other studies indicate that copepods exhibit selective feeding behavior, often distinguishing between natural prey such as phytoplankton and non-food items like microplastics [22]. Differences in feeding strategy in copepods have been linked to differences in microplastic selectivity and ingestion rates. Species that generate a feeding current appear to be able to discriminate based on particle size, shape, and type, generally rejecting microplastics while preferentially consuming algae [22]. The toxicity of plastic debris may come from adsorbed contaminants that become available to zooplankton after ingestion. Toxicity may also come from the leaching of various additives incorporated during manufacturing processes to improve the properties of the plastic. The toxicity of debris that have not adsorbed pollutants is still being debated. Some studies report little or no physical or chemical harm to organisms [23], as fragments in the micrometer range may be readily egested. Other studies report effects, for instance on development and reproduction [24].

In marine and coastal ecosystems where turbulence is very intermittent, zooplankton often experience large fluctuations in flow velocity that compete with their swimming abilities [25]. Experimental measurements indicate that several species, including calanoid copepods, modulate their swimming behavior depending on the intensity of turbulence [26–29]. This response may allow organisms to regulate their vertical position in the water column, which affects how they are transported by currents and helps them to reach or maintain position in favorable locations [10, 11]. Therefore, the rapid response to the hydrodynamic features of the background flow may represent important survival strategies. To transition from being passively transported by the flow to being able of directed motion when turbulence is not too strong, zooplankton organisms must propel themselves with sufficient force to overcome random advection by turbulence or directed transport by currents. In calanoid copepods, this capability is achieved via frequent jumps [26, 27, 30]. When performing jumps, copepods reach velocities that are larger than the turbulent velocity fluctuations in typical oceanic conditions. Behavioral incapacitation caused by pollution may reduce the frequency and amplitude of jumps [31], thereby limiting the ability of copepods to navigate in their environment.

In this study, we focus on the effects of contamination of the calanoid copepod *Eurytemora affinis* by the smallest size fractions of microplastics (5-10 $\mu$m). *E. affinis* represents the most abundant meso-zooplankton in the low to medium salinity zone of most European and North-American estuaries [32]. As a filter-feeder species, it is able of prey selectivity [33] but does ingest microplastics [34]. We recorded the behavior of thousands of unexposed and exposed copepods to polyethylene microfragments in a large measurement volume (20 cm $\times$ 15 cm $\times$ 10 cm). We performed measurements in calm water and in the presence of turbulence to quantify how microplastic ingestion affects their ability to move in hydrodynamic conditions representative of their natural environment.

The transport of particles in turbulence is most naturally described in the Lagrangian frame of reference, where each particle is followed individually as it moves through space and time. Three-dimensional trajectories of tracer particles seeded in the flow and recorded from multiple cameras have been used for a number of years in turbulence research to calculate the Lagrangian properties of turbulent flows [35, 36]. The technique was also used to study the motion of small organisms such as flying insects or copepods [27, 37]. In this study, we used an implementation of such technique, called time-resolved 3D Lagrangian particle tracking velocimetry (3D-LPT). We recorded simultaneously and at high frequency (360 Hz) the motion of thousands of flow tracers and copepods over long time intervals. This allowed us to decouple the contribution of behavior from the contribution of transport by turbulence. We obtained a robust database containing millions of copepod coordinates, from which we isolated a large number of jumps that otherwise would be almost indistinguishable from turbulence fluctuations.

The article is organized as follows. First, we describe the experimental setup, which consists of a 160 L tank in which turbulence is sustained by the motion of two oscillating grids. The setup generates turbulence at intensities close to that experienced by copepods in energetic environments such as estuaries. Then, we describe the measurement technique and the necessary steps to reconstruct the trajectories of tracers and copepods. Particular attention is paid to the method developed to separate copepod trajectories from flow tracer trajectories. We present our experimental results on the relative velocity of copepods with respect to the underlying flow. We characterized the amplitude, duration and frequency of jumps in calm water and in turbulence. Finally, we discuss the response of copepods to turbulence and to microplastics and the implication of behavioral impairments in their ecology.

## Materials and methods

### Copepod cultures

Copepods (approximately 600,000 juveniles and adults) and microalgae (*Rhodomonas baltica* and *Isochrisis galbana*) were obtained from the large-scale cultures maintained at the public aquarium Nausicaá (Boulogne-sur-Mer, France) by the group of S. Souissi. Copepods were grown over a period of several weeks in two transparent tanks (1000 L each) under controlled conditions. Copepods, algae and sea water were shipped overnight from Nausicaá to the Institut de Mécanique des Fluides de Toulouse (IMFT). Microalgae were cultivated at IMFT to feed the copepods during the experimental campaign, which lasted three to four weeks in March 2022.

### Turbulence generation

**Fig. 1:**
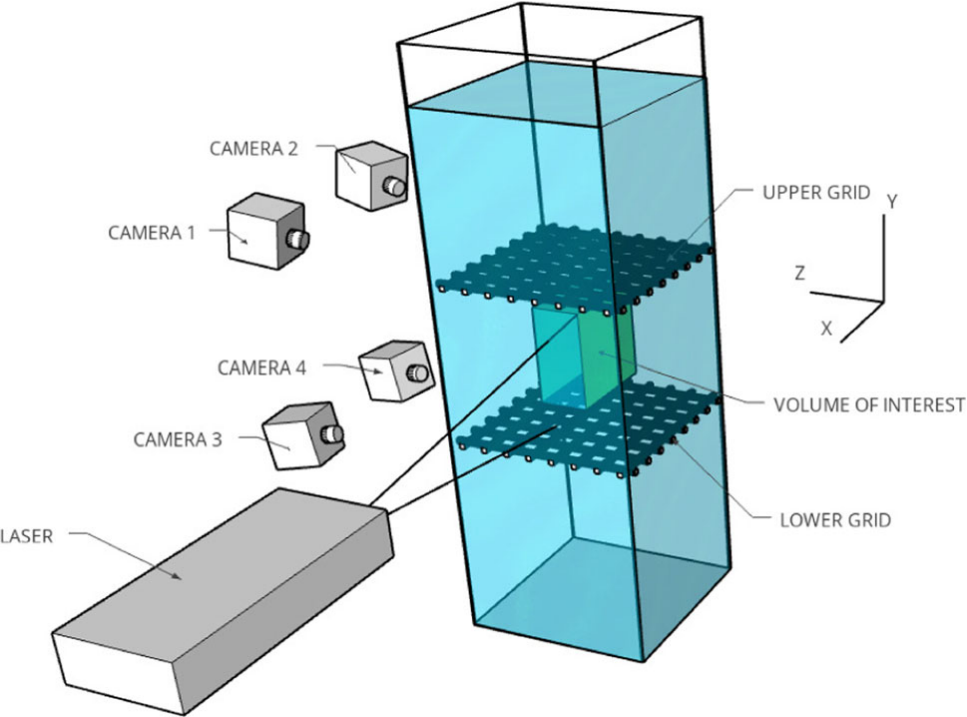
Sketch of the experimental setup. Four synchronized cameras record copepods and flow tracers from one side of the tank. The measurement volume (highlighted in green) is located in the middle of the tank, at equal distance from the two oscillating grids and the walls. Illumination is provided by a green laser located on one side of the tank

Measurements were conducted at IMFT in a rectangular glass tank filled with water at salinity 15 and at 18 $^\circ$C. The dimensions of the experimental tank are 0.4 m (W) $\times$ 0.4 m (D) $\times$ 1.2 m (H) (Fig. 1). We prepared brackish water by dissolving commercial salt (Instant Ocean) in distilled water at a concentration of 15 g/L (density $\rho _f = 1.01$ g/cm$^{3}$). The water was then filtered through 200 nm cartridges to remove impurities responsible for light scattering and attenuation.

Turbulence was generated by the vertical oscillations of two horizontal grids located 40 cm apart. The water level was set at 1.1 m from the bottom of the tank. The distance between the free surface and the average position of the upper grid was 10 cm. The grids are made of square bars of width *M*/5, with $M = 5$ cm the grid mesh. This results in a grid solidity equal to 36 %. This value is close to the solidity of grids often used in wind tunnels to generate homogeneous isotropic turbulence [38] and below the value of 40 % in order to minimize secondary flows [39]. The grids are mounted on a motorized linear slide. They oscillate at a frequency $f = 2$ Hz with a maximum stroke $S = 80$ mm, generating sustained turbulent flow conditions. The forcing is characterized by a stirring Reynolds number $Re = fS^2/\nu _f = 12800$, where $\nu _f$ is the kinematic viscosity of the salty water. The grids were set in motion 30 min before any measurement to ensure a fully developed and statistically steady-state turbulence.

### Microplastic exposure

**Fig. 2:**
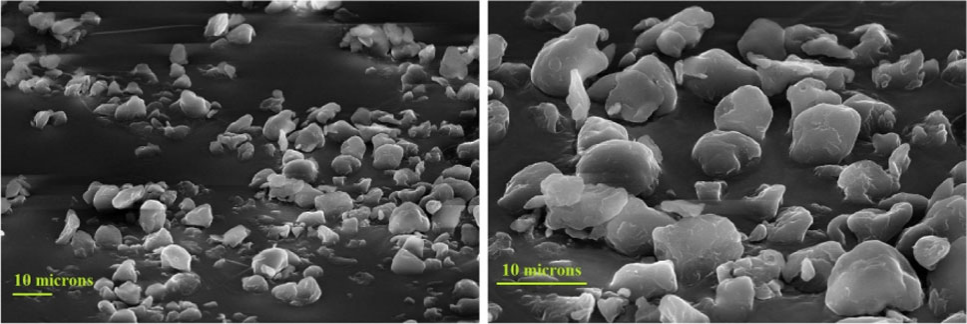
Scanning electron microscopy images of the microplastics used in this study, at two different magnifications

Most plastic microfragments found in the environment are rugged, irregularly shaped polyethylene and polypropylene debris [20], whereas laboratory measurements often use smooth, spherical polystyrene microbeads. In this study, we used ground polyethylene fragments. We observed them with scanning electron microscopy (SEM) to verify their non-spherical appearance. We also conducted morphometric analysis to determine the particle size distribution and shape statistics. The fragments have a typical size of 6-8 $\mu$m and are of various shapes and colors (crushed debris) (Fig. 2). The mean and standard deviation of the particle size distribution are 7 $\mu$m and 2 $\mu$m, respectively. Their shape is characterized by a mean sphericity equal to 0.87 and a mean aspect ratio of 0.7.

Twelve hours before measurement, approximately 60,000 copepods were gently transferred from the cultures to an exposure tank containing 160 L of water at salinity 15 and at temperature maintained between 15 $^\circ$C and 18 $^\circ$C. We used a glass tank to prevent microplastics from attaching to the walls. We added 48 mg of microplastics in order to reach a nominal concentration of 300 $\mu$g/L, which is representative of values observed in polluted zones such as estuaries [40]. Continuous gentle agitation was provided to keep the microplastics homogeneously suspended. Copepods were not fed during exposure to avoid complex interactions between microplastics and microalgae. We also prepared a batch of control (unexposed) copepods using the very same protocol, with the exception that we did not add microplastics to the exposure tank.

Before each measurement, we gently transferred copepods from the exposure tank to the experimental tank and waited an hour for the copepods to acclimate. The concentration in the experimental tank was approximately 400 copepods per liter, which is similar to values observed in estuaries [41] and large enough to obtain reliable statistics. Only adults and late-stage copepodites were considered, because younger developmental stages do not have the swimming capabilities of adults and therefore cannot efficiently self-propel in turbulence.

We recorded the behavior of exposed and unexposed copepods in still water and in turbulence. For each experimental condition, we recorded four consecutive sequences of 40 s each (i.e., 80 grid oscillations). We conducted experiments twice, using new copepods from the culture. In total, we obtained 32 sequences of 40 s each. We checked that the copepods were living and actively swimming at the end of the measurements. In addition, copepods were sampled randomly from the experimental tank and checked individually under the microscope after every measurement. No damage was observed.

### Image acquisition

The measurement volume is rectangular with dimensions 200 mm $\times$ 150 mm $\times$ 100 mm in the *X*, *Y* and *Z* directions, respectively (*Y* being the vertical direction). The origin of the coordinate system lies at the center of the measurement volume. The measurement volume is located at equal distances from the average position of the grids and midway between the walls of the tank. The dimensions of the volume make it possible to track a large number of copepods over a long time. The large distance (more than 10 cm) between the edges of the volume and the walls of the tank prevents confinement effects on the flow. Copepods in the measurement volume were always far from the walls.

Images of flow tracers and copepods were recorded simultaneously using a stereovision system composed of four synchronized Phantom VEO 640 L cameras (Vision Research). The recorded images have a resolution of 2560 by 1600 pixels at $2^{12}$ gray levels. The cameras were fitted with 100 mm macro-lenses (Zeiss) mounted on LaVision Scheimpflug V3 adapters with an aperture set to *f*/11. They were tilted by approximately 20 $^\circ$ from the direction perpendicular to the tank wall, toward the region of interest. Considering Scheimpflug conditions, the optical axis of the camera lenses was directed toward the center of the illuminated volume.

Illumination was provided by a high-power dual cavity laser (Photonics Industries DM60-527-DH) providing 2 $\times$ 60 mJ per pulse at 1 kHz. Knife-edge masks were inserted in the light patch to produce sharp boundaries of the illuminated volume. A mirror was placed on the side of the tank opposite to the laser to increase light intensity within the measurement volume [42]. The laser and the four cameras were triggered by a signal sent by a LaVision Programmed Timing Unit driven by the DaVis acquisition software. The whole system operated at an acquisition frequency of $f_a = 360$ Hz which is fast enough to resolve the velocity of copepods during jumps. The time scale $1/f_a$ is more than an order of magnitude smaller than the Kolmogorov time scale of turbulence.

To characterize the flow, the water was seeded with spherical polyamide particles (Orgasol^®^) at a concentration of approximately 3.10$^4$ particles per liter. These particles have a diameter $d_p = 20\,\mu$m and a density $\rho _p = 1.03$ g/cm$^3$. They are characterized by a very low Stokes number $St = (1/18)(\rho _p/\rho _f)(d_p/\eta )^2$, where $\eta$ is the Kolmogorov length scale of the flow. *St* is always much lower than $\mathcal {O}(10^{-4})$. The settling velocity of tracer particles is given by the Stokes law as $gd_p^2(\rho _p-\rho _f)/18\mu _f$, where *g* is the gravitational acceleration and $\mu _f$ the dynamic viscosity of the fluid. This reference velocity is close to 5.10$^{-6}\,$m/s, which is much lower than the typical flow velocity fluctuations in our measurements. Therefore, we consider that these particles passively follow the fluid motion.

### Image processing

**Fig. 3:**
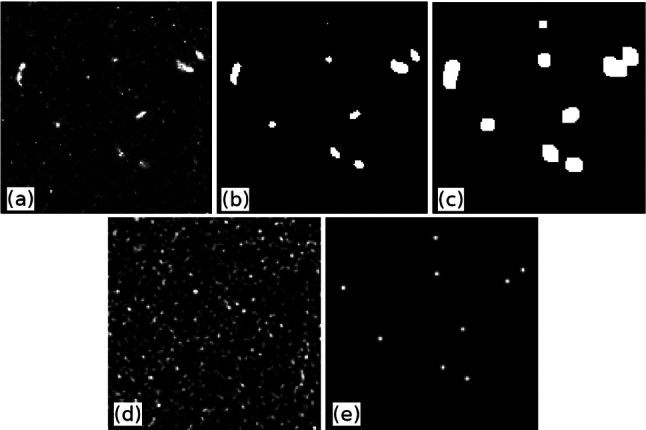
Illustration of the image processing steps on a small region of an image (100 $\times$ 100 pixels). **a** Raw image obtained after subtracting from each pixel its minimal intensity observed over the whole sequence. **b** Binary image of copepods. **c** Copepod mask image. **d** Image of tracer particles obtained by subtracting the copepod mask image from the raw image. **e** Pseudo-copepod image obtained after replacing the silhouette of a copepod with a spherical blob. The pixel intensity within the blob follows a 2D Gaussian distribution

We first removed background elements such as light reflections or immobile objects by subtracting from each pixel its minimal intensity observed over the whole sequence. We obtained the so-called raw images (Fig. 3a) from which we created two sets of images: one set to reconstruct the trajectories of copepods, and a second set to reconstruct the trajectories of tracers. The procedure consists of the following steps: (i) detection of copepods in the raw images, (ii) removal of detected copepods from the raw images to generate images containing flow tracers only and (iii) generation of pseudo-copepod images containing the smoothed silhouettes of the copepods only. The two sets were then processed independently using the Shake-The-Box (STB) algorithm. The procedure is described in more detail below.

#### Identification of copepods

Copepods in the images appear as white blobs that are larger and brighter than the surrounding polyamide tracer particles (Fig. 3a). The average size and pixel intensity of copepods in the grayscale images are 10 pixels and 2800, respectively. The average size and pixel intensity of tracers are 3 pixels and 300, respectively. We applied a local median filter of size $5 \times 5$ pixels to remove the high spatial frequency signal of flow tracers and to improve the signal of the copepods relative to the polyamide particles. Copepods were subsequently identified using thresholding: pixels whose value exceeded a predefined threshold of 500 were categorized as belonging to a copepod. The resulting binary images (Fig. 3b) were used to generate both pseudo-copepod images and copepod mask images.

#### Images of tracer particles

Bright objects in the binary images were slightly enlarged by morphological dilation using a $5 \times 5$ flat structuring element to obtain the copepod mask images (Fig. 3c). The goal of the morphological dilation was to ensure that each copepod is completely included in the mask. The copepod mask images were then subtracted from the raw images to create the tracer particle images (Fig. 3d). These images underwent additional processing to facilitate the detection of tracers particles by the tracking algorithm. We performed a contrast stretching transformation that reduced the intensity of the background and increased that of the particles. A Gaussian smoothing with a $3 \times 3$ filter was then used to homogenize the particle shape. Finally, the subtraction of a fixed pixel intensity and the multiplication by a fixed gain enhanced the gray-level dynamics.

#### Pseudo-copepod images

Because the STB algorithm requires knowledge of the global shape of the objects being tracked, an Optical Transfer Function (OTF) is necessary [43]. The OTF plays a key role in modeling how particle images form across multiple camera views. It characterizes the optical response to a point source and therefore directly affects how particles appear on the sensor. An accurate OTF model is essential for the projection and reprojection steps of the STB algorithm, where predicted particle positions are projected onto the image planes and compared to the actual intensity patterns. Any discrepancy between the modeled and actual OTF results in systematic errors in particle triangulation and can lead to increased residuals during the iterative “shaking” process.

However, copepods have anisotropic shapes. Their silhouette in the images varies strongly depending on their orientation and position, making it difficult to define a reliable OTF. To address this issue, we processed the copepod images to make copepods appear as spherical particles with isotropic pixel intensity before using the STB algorithm. Each object in the binary images was replaced by a spherical blob centered on the object center of mass. The pixel intensity of the blob follows a 2D Gaussian distribution with a standard deviation of one pixel (Fig. 3e).

Additionally, the relatively small number of copepods compared to tracer particles in the measurement volume does not allow for an accurate estimation of a specific pseudo-copepod OTF during the self-calibration step. Instead, the STB algorithm uses the well-characterized OTF of tracer spherical particles for 3D tracking of the pseudo-copepods. The Gaussian parameters are chosen such as the size of the pseudo-copepod image closely matches that of the tracer particles. It should be noted that the camera configuration was chosen with a relatively small angle (20 $^\circ$ to the tank wall) to minimize optical aberrations that could distort the shape of tracer particles in some areas of the camera field of view.

### Trajectory reconstruction

#### Particle tracking

To reconstruct the trajectories of copepods and flow tracers, we processed the tracer images and the pseudo-copepod images using DaVis 10.1 (LaVision GmbH, Göttingen, Germany). The software contains an implementation of the STB algorithm [44, 45] which allows for the tracking of particles at high seeding concentrations. The software also integrates various techniques such as 3D volume self-calibration [46], optical transfer function (OTF) [47], and an iterative reconstruction method for the volumetric particle distribution known as IPR [43].

Shake-The-Box (STB) is a Lagrangian particle tracking algorithm that predicts the spatial locations of seeding particles within the flow by extrapolating the positions at the next time step from existing data. This method maximizes the use of temporal and spatial information extracted from the images. Particle positions and velocities are first extrapolated using Newton’s equations of motion from previously known trajectories using a predictive Wiener filter [45, 48]. Then, particle positions are corrected through iterative optimization in order to minimize the residuals between the projected particle images and the actual intensity distributions recorded by the cameras. This “shaking” process refines particle positions in each frame by iteratively adjusting them to best match the observed intensity patterns, thereby enhancing both tracking accuracy and particle detection rate.

#### Camera calibration

We calibrated the cameras using images of a reference object with target points of known coordinates. The reference object consists of a two-level calibration on which reference points are evenly distributed (309-15 3D calibration plate from LaVision). The calibration plate was immersed in the experimental tank in such a way that it aligned with the *X*- and *Y*-axes of the coordinate system. A computer-controlled translation device (1 $\mu$m accuracy) was used to move the calibration plate along the *Z*-axis. The plate was imaged by the four cameras at eight different locations within the measurement volume. The DaVis software identified the pattern of the calibration plate and computed the mapping function between the 2D images and the laboratory coordinate system at each position along the *Z*-axis. Interpolation of these 2D mapping functions at each calibration point yielded a 3D calibration function with a maximum error close to 0.2 pixel.

Errors below 0.1 pixel are required to effectively apply the STB algorithm [44, 46, 47]. Therefore, we also performed an additional dynamic calibration based on the images of moving tracer particles. A set of 100 uncorrelated images containing only tracer particles was used to perform the self-calibration procedure [46, 49]. This iterative process adjusts the calibration function to minimize the discrepancy between actual and re-projected images. This leads to more accurate volumetric spatial calibration and optical transfer function. In our measurements, self-calibration reduced the discrepancy down to an average value of 0.02 pixels.

During normal cruising, the velocity of copepods is low and the relative error on determining the position results in a relative error in the displacement (yielding velocity) that is larger than during jumps. This quantitative error on the exact location of the copepod center of mass is essentially related to its complex shape and interaction with light. The major contribution to the error is associated with the pre-processing of images when copepods are substituted to a Gaussian spherical object before the Shake-The-Box algorithm is applied. We can consider that the error is lower than 0.1 pixel which with frequency acquisition of 360 Hz yields an upper bound of 3.6 mm/s for the absolute error of the velocity. This is ten times lower than the threshold we use to detect jumps and provides at least ten time steps within a typical jump duration. It has been shown [48] that time-resolved tracking measurements help to reduce the uncertainties on the location of particles and then reduced errors on the velocity measurements.

#### Trajectory post-processing

Reconstructed trajectories of tracer particles and copepods were post-processed to remove spurious detections and to compute particle velocity and acceleration. Only trajectories with a minimum duration of one second and a minimum path length of $5\,$mm were considered. Shorter trajectories typically result from impurities in the water or intermittent reflections on the inner surface of the glass tank. Trajectories were smoothed with a third-order Savitzky–Golay filter with a span of 11 frames to improve the measurement of velocity and acceleration without adversely affecting high-velocity events caused by jumps or turbulent velocity fluctuations [26]. Velocity and acceleration were computed directly from the fitted polynomial.

### Trajectory analysis

#### Relative velocity

**Fig. 4:**
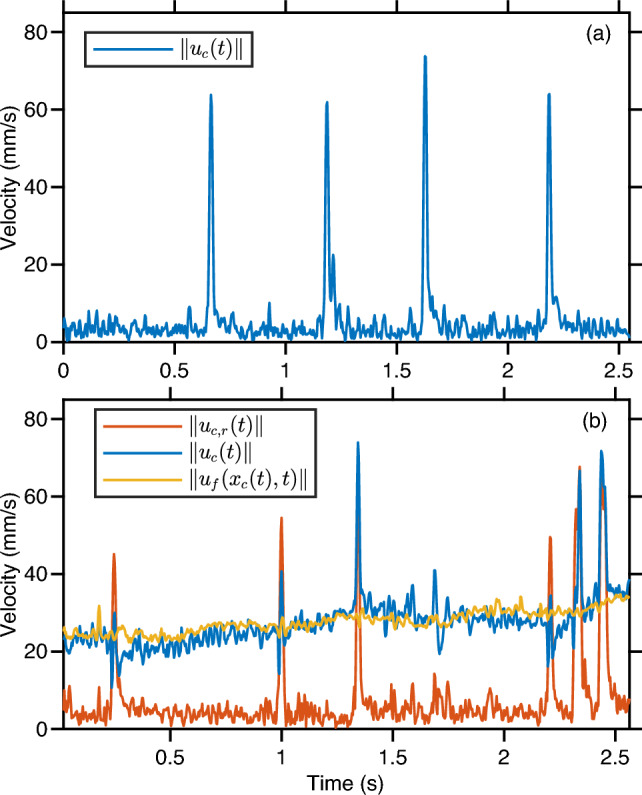
Typical time series of the magnitude of copepod velocity $\Vert \boldsymbol{u}_c(t)\Vert$ for copepod swimming in: **a** calm water; **b** a turbulent flow. For the turbulent case, the magnitude of the flow velocity at the copepod position, $\Vert \boldsymbol{u}_f(\boldsymbol{x}_c(t),t)\Vert$, as well as the magnitude of the copepod relative velocity, $\Vert \boldsymbol{u}_{c,r}(t)\Vert$, are also shown

We used the velocity of tracers to calculate the relative velocity of copepods with respect to the underlying flow. The relative velocity is defined as $\boldsymbol{u}_{c,r}(t) = \boldsymbol{u}_c(t) - \boldsymbol{u}_f(\boldsymbol{x}_c(t),t)$, where $\boldsymbol{u}_c(t)$ is the velocity of the copepod and $\boldsymbol{u}_f(\boldsymbol{x}_c(t),t)$ is the local instantaneous flow velocity interpolated at the position $\boldsymbol{x}_c(t)$ of the copepod. The interpolation procedure uses a Delaunay triangulation of the tracer velocities within the measurement volume to estimate the flow velocity at the position of each copepod. Figure 4 shows a typical time series of the magnitude of $\boldsymbol{u}_{c}(t)$, $\boldsymbol{u}_f(\boldsymbol{x}_c(t),t)$, and $\boldsymbol{u}_{c,r}(t)$ from the trajectory of a copepod swimming in calm water (Fig. 4a) and in turbulence (Fig. 4b). In still water, the relative velocity coincides to the absolute velocity, since the flow velocity is zero.

**Fig. 5:**
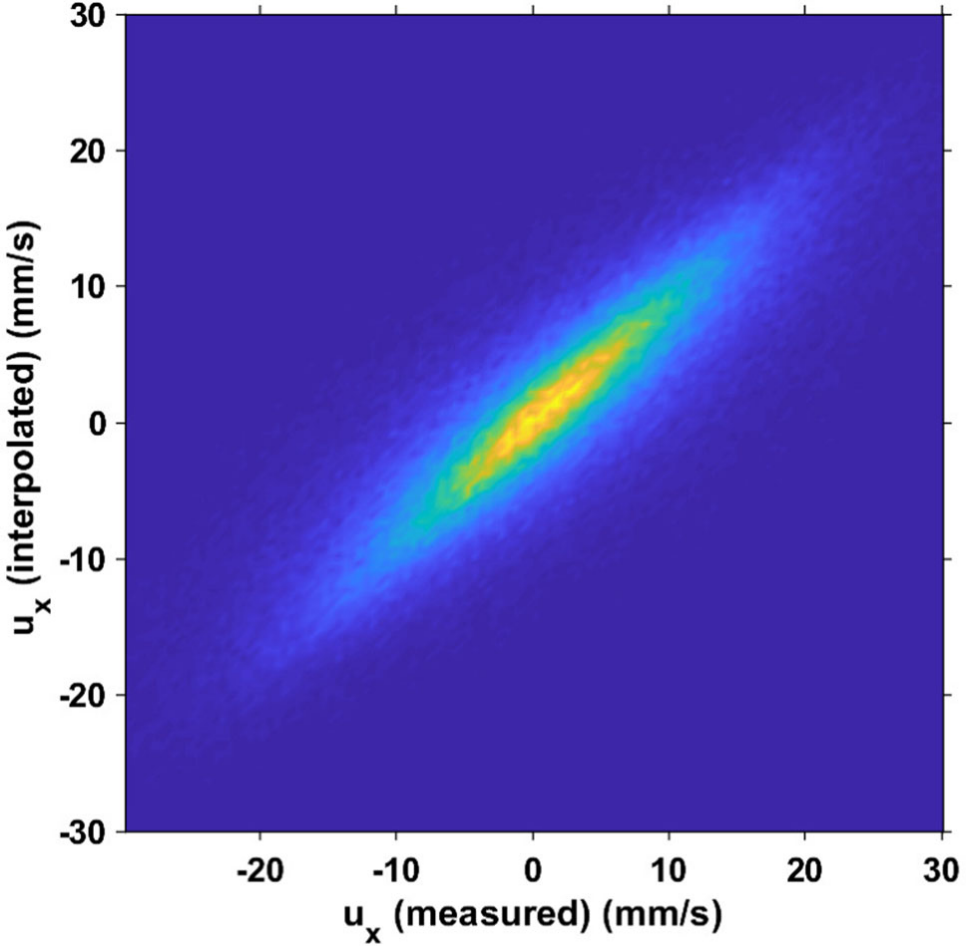
Correlation between the *X* component of the measured velocity and the interpolated velocity within the measurement volume. The correlation coefficient is 0.85

The number of tracer particles in the measurement volume was estimated from the number of tracer trajectories reconstructed by the STB algorithm. It is $\mathcal {O}(10^5)$, leading to an average separation distance between tracers on the order of 3 mm. This corresponds approximately to 8 times the Kolmogorov length scale and 3 times the typical size of copepods. Therefore, local sampling is sufficient to provide a reliable spatial interpolation of the fluid velocity at any location in the measurement volume. To verify that the estimation of $\boldsymbol{u}_f(\boldsymbol{x}_c(t),t)$ is correct, we interpolated the fluid velocity at the position of $10^4$ randomly chosen tracer particles, using the exact same interpolation algorithm. When interpolating the velocity at the position of a tracer particle, we discarded the position and velocity of the query point (i.e., the tracer) from the positions and velocities of the sample points (i.e., all the other tracers). We compared the measured flow velocity to the velocity interpolated at the location of the particles. The value of the correlation coefficient (0.92 and 0.89 for the *Y* and *Z* components of the velocity, respectively) indicates that the procedure provides an accurate interpolation of the flow velocity and that masking copepods to extract tracer positions in the images does not negatively influence the quantification of the flow field. Figure 5 shows the correlation for the *X* component, which is slightly lower (0.85).

#### Jump detection

**Fig. 6:**
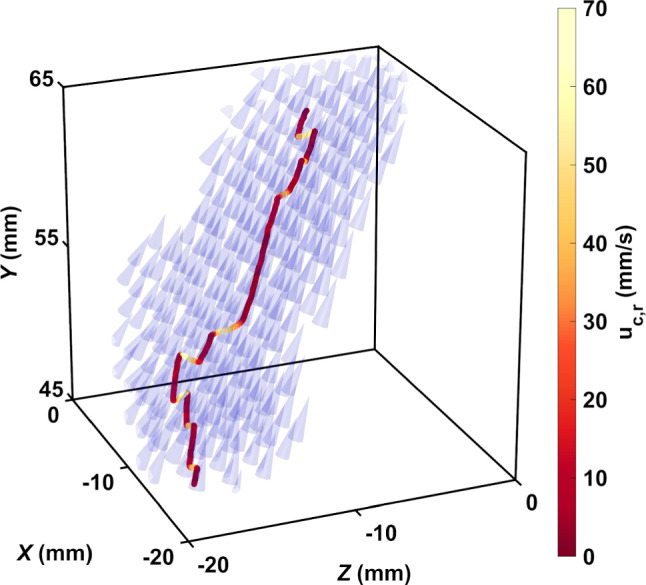
Trajectory of a copepod swimming in turbulence, color-coded with the magnitude of its relative velocity with respect to the flow to emphasize the contribution of motility, and instantaneous flow field along the trajectory. The length of the cones is proportional to the flow velocity

In calm water, many species of calanoid copepods swim by alternating periods of slow cruising motion with frequent relocation jumps [27, 50]. Cruising motion is characterized by slow velocities on the order of a few millimeters per second, while jumps can reach tens of millimeters per second. In turbulence, cruising is indistinguishable from transport by the flow, but jumps remain clearly visible because their amplitude (up to 150 mm/s in our measurements) is larger than the typical flow velocity fluctuations (approximately 12 mm/s RMS in our measurements). Jumps appear as intermittent events of high amplitude in the relative velocity time series (Fig. 4). As a result, copepods are able to depart from the underlying flow. Figure 6 shows the trajectory corresponding to Fig. 4b and the flow velocity field around the copepod. The motion of the copepod follows that of the flow, except during jumps.

**Fig. 7:**
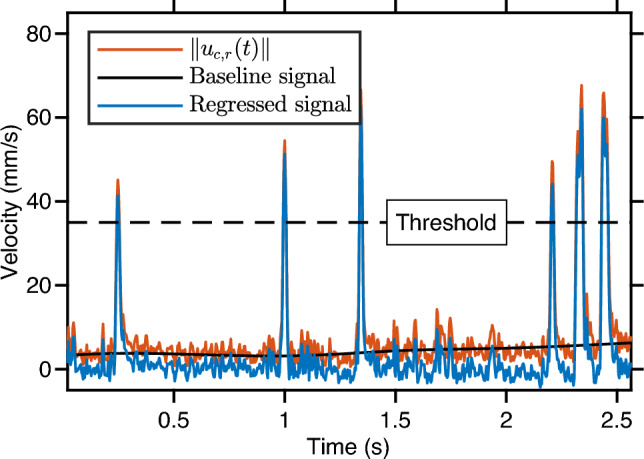
Time series of the magnitude of the copepod relative velocity, showing the raw signal and the regressed signal after wavelet denoising

We extracted jump events from the relative velocity time series as follows. First, we applied a wavelet filter to reduce fluctuations due to noise while preserving peaks. Then, for each trajectory, we subtracted the baseline of the smoothed time series and identified all local maxima. Any local maximum above a threshold of 35 mm/s is considered a jump. Figure 7 shows the result of the baseline subtraction on the trajectory described earlier. The amplitude of a jump is the value of the regressed signal at the corresponding local maximum. Its duration is the time between the first local minima on both sides of the local maximum.

## Results

**Fig. 8:**
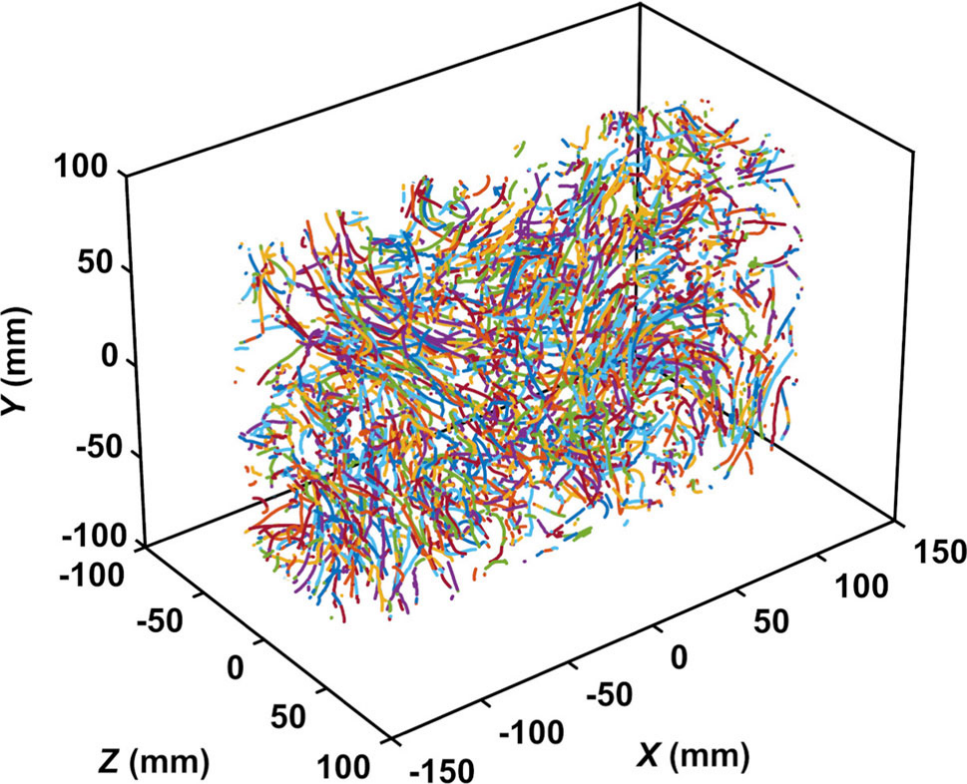
Example of trajectories of copepods swimming in turbulence during 2 s. Copepods appear to be homogeneously distributed within the entire measurement volume. Large-scale structures of the underlying turbulent flow are also visible. Because the cruising velocities of copepods are low, on the order of a few millimeters per second, they do not contribute significantly to transport in turbulence. Therefore, a copepod is transported by the flow until it jumps. While jumping, it reaches velocities that are larger than the flow velocity fluctuations, and consequently it deviates from the flow streamlines. However, because the distance traveled during jumps (a few millimeters) is small compared to the size of the measurement volume, jumps are difficult to distinguish. As a result, trajectories of copepods may look similar to trajectories of flow tracers

We show in Fig. 8, as an illustration of the data gathered during the measurements, the trajectories of unexposed copepods swimming in turbulence over a 2 s duration. We obtained $\sim 2.10^7$ data points for copepods in calm water and more than $4.10^7$ for copepods in turbulence. For each of the four sequences associated to each experimental condition, the number of detected copepods remained roughly constant during recording. The mean trajectory duration is 16 s for copepods swimming in calm water and 2 s for copepods in turbulence. The trajectory duration is shorter in turbulence because the time spent by the copepods within the observation volume is much shorter than in calm water due to convective transport by the large scales of the flow. Broken trajectories are also more likely to occur because of the difficulty in tracking copepods that jump on top of strong flow fluctuations.

**Table 1:** Flow parameters in the measurement volume. $\sigma _{u_i}$ is the root-mean-square of the velocity fluctuation ($i = x,y,z$). $\varepsilon$ is the space- and time-average turbulent kinetic energy dissipation rate. $\eta = (\nu ^3 / \varepsilon )^{1/4}$ and $\tau _{\eta } = (\nu / \varepsilon )^{1/2}$ are the Kolmogorov length and time scales, respectively

$\sigma _{u_x}$	$\sigma _{u_y}$	$\sigma _{u_z}$	$L_x$	$L_y$	$L_z$	$\varepsilon$	$\eta$	$\tau _{\eta }$
(mm/s)	(mm/s)	(mm/s)	(mm)	(mm)	(mm)	(mm$^2$/s$^{3}$)	(mm)	(s)
10.8	13.3	11.7	14	19	15	65	0.35	0.13

### Flow parameters

Flow parameters in the measurement volume are given in Table 1. They were computed from Eulerian statistics by binning the Lagrangian information obtained from the STB algorithm onto a fixed 3D grid located in the center of the measurement volume. The grid measures 66 voxels in the *X* direction, 50 voxels in the *Y* direction, and 20 voxels in the *Z* direction. The binning yields at each time step an instantaneous 3D Eulerian velocity field $\boldsymbol{u}(\boldsymbol{x},t)$ with a 3 mm spatial resolution.

Fluctuating velocity components are defined as $u'_i(\boldsymbol{x}) = u_i(\boldsymbol{x},t) - {U_i}(\boldsymbol{x})$ where ${U_i}$ is the temporal mean velocity field and $i = x,y,z$. The root-mean-square of the velocity fluctuation is given by $\sigma _{u_i} = \langle u'^2_i \rangle ^{1/2}$ where $\langle \cdot \rangle$ denotes the spatial and temporal average over the measurement volume and the 40 s sequence duration. These one-point statistics indicate that the 3D turbulent flow generated by the two active grids is roughly isotropic with $\sigma _{u_x} \approx \sigma _{u_z}$ and $\sigma _{u_y} \approx 1.18\,(\sigma _{u_x} + \sigma _{u_x}) / 2$, $u_y$ being the vertical velocity component perpendicular to the grids. Similar observation was reported in [51] using a similar turbulence generator. Additionally, the flow can be considered satisfactorily homogeneous as the standard deviation of the turbulent kinetic energy does not exceed 10 % of its spatially averaged mean value.

The integral length scale of the flow is defined as the integral of the longitudinal fluctuating velocity correlation $L_i = \int \langle u'_i(\boldsymbol{x} + r \boldsymbol{e}_i,t) u'_i(\boldsymbol{x},t) \rangle / \langle u'^2_i \rangle dr$, $i = x,y,z$ and $\boldsymbol{e}_i$ is a unit vector. They are comparable in the *X* and *Z* directions ($L_x \sim 14$ mm and $L_z \sim 15$ mm) but slightly larger in the vertical direction perpendicular to the grids ($L_y \sim 19\,$mm). The turbulent kinetic energy dissipation rate $\varepsilon$ is estimated from the Eulerian second-order longitudinal velocity structure function $(S_2(r) =\langle \delta u^2(r) \rangle )$ where $\delta u(r) = \left[ u'(\boldsymbol{x} + \boldsymbol{r},t) - u'(\boldsymbol{x},t) \right] \cdot \boldsymbol{r} / r$ is the longitudinal velocity increment between points of the flow separated by $\boldsymbol{r}$. For isotropic turbulence and a distance *r* in the inertial range, $S_2(r) = C (\varepsilon r)^{2/3}$ with $C \approx 2.1$ [52]. This estimate was compared to the relation $\varepsilon \simeq u_{rms}^3/L$ where the integral scale *L* is taken as the average of $L_x$, $L_y$ and $L_z$. Both methods yield similar results. The Kolmogorov length and time scales, respectively, defined as $\eta = \left( \nu / \varepsilon \right) ^{1/4}$ and $\tau _\eta = \left( \nu / \varepsilon \right) ^{1/2}$, are directly obtained from $\varepsilon$ estimated from $S_2(r)$.

The magnitude of the dissipation rate of the turbulent kinetic energy in our laboratory-scale turbulent flow ($\varepsilon \simeq 65\,$mm$^2$/s$^{3}$) is larger than values measured in the open ocean under normal conditions, where $\varepsilon$ typically ranges from 10$^{-2}$ mm$^2$/s$^3$ to 1 mm$^2$/s$^{3}$ in the upper 100 m [53–55]. It is however comparable to values measured in estuaries where $\varepsilon$ reaches 10$^{2}\,$mm$^2$/s$^{3}$ [56, 57]. The typical fluctuating velocity in our measurements ($\sigma _{u} \simeq 12\,$mm/s) is comparable to values observed in field measurements [58, 59].

### Microplastic ingestion

**Fig. 9:**
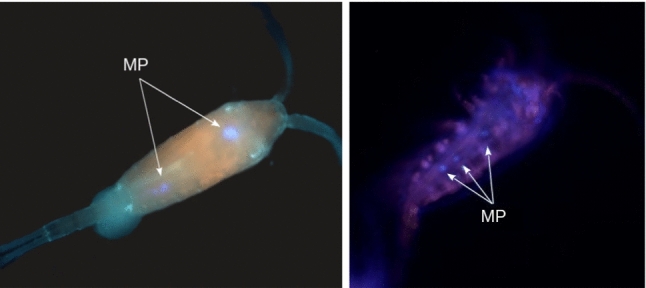
Fluorescence microscopy observation of copepods exposed to microplastics (MP). Plastic fragments stained with Nile Red are visible in their digestive track

A sample of copepods was taken from the exposure tank before each measurement. Copepods were immediately placed in ethanol (70 %) and then washed with brackish water at salinity 15 to remove any plastic fragments adsorbed on their cuticle. They were transferred to a beaker filled with 20 ml of water at salinity 15. To verify for microplastic ingestion, Nile Red (Sigma-Aldrich, N3013) was added to the solution to reach a concentration of 15$\,\mu$g/L [34]. After 10 min, the copepods were washed again to remove the stain solution and placed under a fluorescence microscope with a DAPI filter (355-394 nm). Observation at 20$\times$ and 40$\times$ magnification revealed the presence of microplastic particles in the digestive track of the copepods (Fig. 9).

### Effect of microplastics on copepod behavior

#### Control copepods

**Fig. 10:**
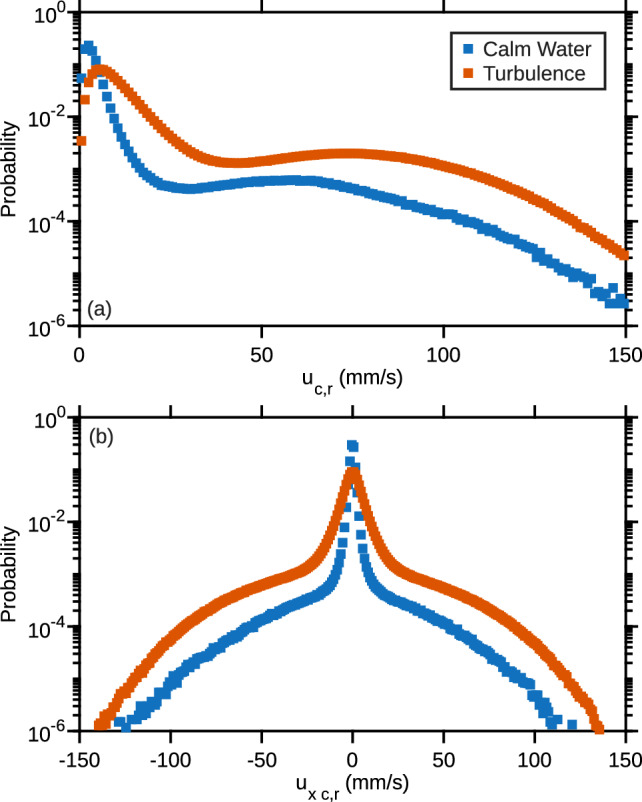
Results obtained with control (unexposed) copepods. **a** PDF of the magnitude of the relative velocity in calm water (blue) and in turbulence (red). **b** PDF of the component along *X* of the relative velocity in calm water (blue) and in turbulence (red). Data for all sequences and the two replica have been combined

We start our analysis by considering the behavior of unexposed copepods swimming in calm water and in turbulence. Figure 10 shows the probability density function (PDF) of the magnitude of the relative velocity $\Vert \boldsymbol{u}_{c,r} \Vert$ and the PDF of its component $u_{x\,c,r}$ along the *X*-axis. In calm water, the relative velocity corresponds to the absolute velocity, as the flow velocity is zero. Data for all sequences and the two replica have been combined.

In still water, the shape of the PDF is consistent with previous experimental observations in the same species [26]. Many calanoid copepods swim by alternating periods of slow swimming (cruising) and frequent relocation jumps [50]. Cruising derives from the creation of feeding currents accomplished by the high-frequency vibration of the cephalic appendages [60]. Relocation jumps originate from the repeated beating of the swimming legs and result in a sequence of high-velocity events leading to an intermittent motion [50]. The largest probabilities in Fig. 10 correspond to velocities below 10 mm/s associated with cruising. The tail of the distribution corresponds to jumps, with velocities up to 150 mm/s. Large velocities occur more frequently than in the case of a Gaussian distribution. The PDF of $u_{x\,c,r}$ shows that there is no preferred direction of motion (similar results were obtained for the two other components). We note that the amplitude of the relocation jumps observed in our measurements is smaller than the amplitude of escape jumps that copepods perform in response to threats such as nearby hydrodynamic disturbances or sudden changes in light intensity [61]. During escape jumps, copepods accelerate up to 2000 mm/s$^{2}$ and reach velocities above 150 mm/s [62].

In turbulence, we observed a decrease in the probabilities of small velocities associated with cruising (below 10 mm/s). Cruising velocities are too small to allow copepods to self-propel in turbulence, and therefore they tend to zero once the flow velocity is subtracted. However, they do not reach exactly zero, which we attribute to errors in the determination of the flow velocity at the location of the copepods, leading to low-amplitude noise in their relative velocity. We also observed a significant increase in the probabilities of larger velocities associated with relocation jumps. We note that the shape of the jumps in the velocity time series (Fig. 4) is comparable to that observed in a previous work in the same species [63]. Jumps are characterized by a sudden increase in $u_{c,r}$ associated with accelerations over 200 mm/s$^{2}$. Velocity then relaxes exponentially due to viscous drag.

**Fig. 11:**
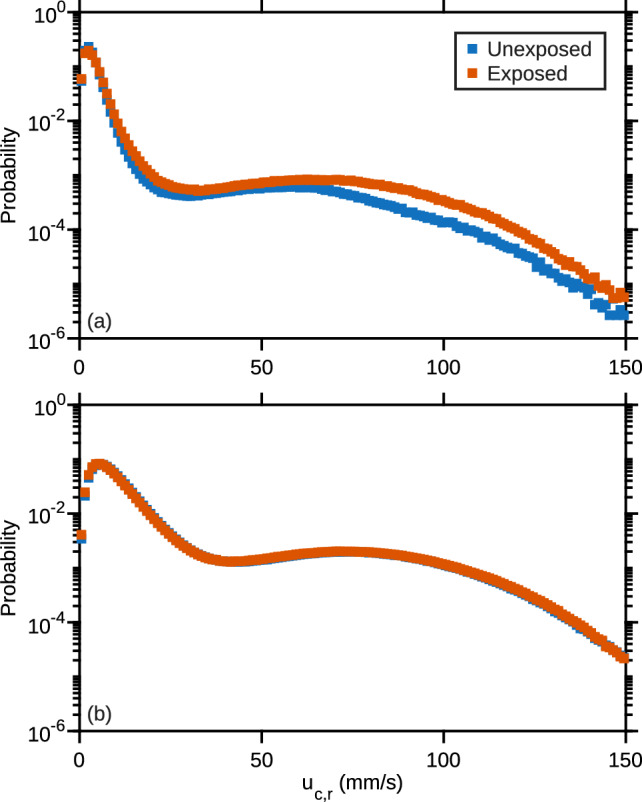
Results obtained with exposed copepods. **a** PDF of the magnitude of the relative velocity of control (blue) and exposed (red) copepods swimming in calm water. **b** PDF of the magnitude of the relative velocity of control (blue) and exposed (red) copepods swimming in turbulence. Data for all sequences and the two replica have been combined

**Table 2:** Jump frequency, amplitude and duration for control and exposed copepods swimming in still water and in turbulence

	Unexposed to microplastics		Exposed to microplastics	
	Calm water	Turbulent flow	Calm water	Turbulent flow
Number of data points	19.10$^6$	43.10$^6$	13.10$^6$	31.10$^6$
Number of trajectories	1671	24489	1540	24187
Number of detected jumps	16.10$^3$	198.10$^3$	19.10$^3$	202.10$^3$
Jump frequency (s$^{-1}$)	1.94	3.64	2.40	3.76
Jump amplitude (mm/s)	69.5 ± 22.0	81.8 ± 26.9	77.2 ± 23.8	82.5 ± 27.0
Jump duration (ms)	30.5 ± 23.7	39.3 ± 30.7	35.1 ± 25.0	40.5 ± 31.9

**Fig. 12:**
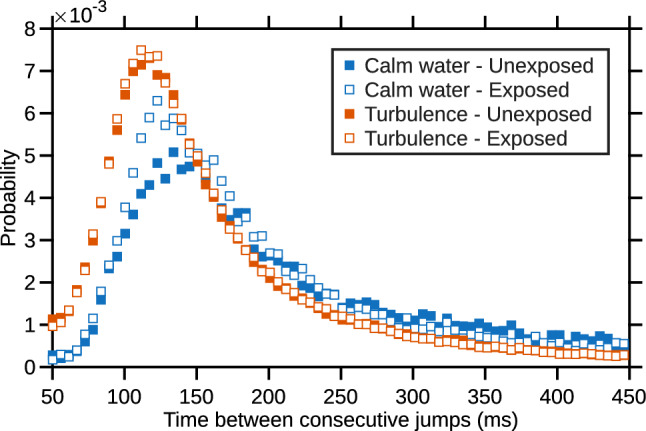
PDF of the separation time between two consecutive jumps for unexposed copepods in calm water (blue, solid markers), unexposed copepods in turbulence (red, solid markers), exposed copepods in calm water (blue, open markers), and exposed copepods in turbulence (red, open markers)

Since we detected a large number of jumps, we can derive robust statistics regarding their amplitude, duration and frequency (Table 2). Jump amplitude increased slightly from 69.5 mm/s in calm water to 81.8 mm/s in turbulence. Jump duration increased slightly from 30.5 ms in calm water to 39.3 ms in turbulence. Jump frequency almost doubled from 1.94 s$^{-1}$ in calm water to 3.64 s$^{-1}$ in turbulence which highlights the behavioral response of copepods to the turbulent environment.

#### Exposed copepods

In calm water, the PDF of $\Vert \boldsymbol{u}_{c,r} \Vert$ shows an increase in the probability of velocities associated with relocation jumps in exposed copepods compared to control copepods (Fig. 11a). This change is mainly due to an increase in jump frequency. Indeed, Table 2 indicates that exposed copepods jump on average 25 % more frequently than unexposed copepods. Jump amplitude increased slightly from 70 mm/s in unexposed copepods to 77 mm/s in exposed copepods. Jump duration increased from 30 ms to 35 ms. The energetic cost associated with active swimming can be quantified by considering the instantaneous power *P*(*t*) per unit mass which is the scalar product of the acceleration and velocity of the copepod along its trajectory. We observe a 40 % increase in the RMS value of *P* when copepods swimming in calm water are exposed to microplastics, confirming that the hyperactivity induced by microplastics ingestion is accompanied by an additional energetic cost ($P_\text {rms}$ for unexposed copepods is 2.53.10$^4\,$mm$^2$/s$^{3}$ and 3.58.10$^4\,$mm$^2$/s$^{3}$ for exposed ones) which may yield tiredness on the long term. In turbulence, the PDF of $\Vert \boldsymbol{u}_{c,r} \Vert$ shows no difference between exposed and unexposed copepods (Fig. 11b). Jump characteristics did not change much (Table 2).

The effects of turbulence and microplastics are clearly visible in Fig. 12 which shows the PDF of the time interval between two consecutive jumps for exposed and unexposed copepods and for both flow conditions. For control copepods swimming in calm water, the PDF shows a broad distribution, meaning that there is no characteristic time scale between jumps. Exposure to microplastics caused an increase in the probability of smaller values, leading to a peak of the PDF at $\approx$ 125 ms. Exposure to turbulence resulted in a marked shift of the distribution toward shorter separation times. In turbulence, the PDF shows no appreciable difference between exposed and unexposed copepods.

## Conclusion

The properties of zooplankton motion have been shaped through evolution by ecological trade-offs, meaning that organisms have evolved swimming strategies to minimize dangerous encounters with predators while maximizing positive encounters with resources and mates [16]. By altering the properties of their motion, pollution can potentially impair these strategies, with effects on downstream ecological processes such as biotic interactions and population spatial distribution. Studying how pollution impairs the behavior of zooplankton therefore gives insight into a range of adverse effects that occur at sublethal level but that may have important ecological consequences [64].

However, the identification and classification of behavioral alterations remain difficult because of the challenges of obtaining robust behavioral data and also because different contaminants seem to induce opposite effects. For instance, laboratory measurements have shown that polycyclic aromatic hydrocarbon, polychlorinated biphenyls, surfactants, and heavy metals at sub-lethal concentrations cause hyperactivity and faster swimming speed in calanoid copepods and cladocerans [17, 65]. On the opposite, exposure to polystyrene microbeads reduces swimming speed in the calanoid copepod *Temora turbinata* [66].

We studied the effects of polyethylene fragments at 300 $\mu$g/L on the motion of the widespread estuarine copepod *Eurytemora affinis* [67]. The same concentration was also used in a recent study examining the effects of multigenerational exposure to microplastics of the same species [68]. We recorded its swimming behavior in calm water to allow comparison with other studies and in turbulence because *E. affinis* is exposed to strong hydrodynamic conditions for most of its life cycle. In calm water, exposure to microplastics for 12 h increased jump frequency by 25 %. Exposure to turbulence increased jump frequency by 90 % in unexposed copepods and 50 % in exposed copepods. In our measurements, the strong response of *E. affinis* to turbulence masked any potential effect of microplastics. Our findings are limited by the biological and physicochemical specificities of the experimental conditions and species. While the exposure duration and concentration were chosen to reflect real-world scenarios, there is clearly a need for further research to fully understand how varying these factors might affect copepod behavior. Future studies to broader the ecological relevance of these findings should incorporate multiple species, different polymer types, and more realistic microparticle coverage such as biofilms. Our findings align with previous studies and highlight the complexity of microplastic impacts. Longer exposure may not change much the response as copepods *E. affinis* have the capacity to release ingested microplastics into their feces after 12 h [19].

Microplastics can alter feeding rates and reduce energy acquisition in copepods [69]. The hyperactivity observed in the present study can incur additional energetic costs, potentially depleting limited energy reserves that are normally allocated to vital processes such as growth and reproduction. More frequent relocation jumps may further compromise energy intake by reducing the time spent by copepods cruising and generating feeding currents. Behavioral changes may also affect encounter rates with predators or mates, leading to elevated predation risk and altered trophic interactions [70, 71]. All together, the lower energy intake combined with extra energy expenditure and impaired biological interactions could significantly reduce copepod fitness [72]. Finally, hyperactivity may disrupt large-scale displacements in the water column if copepods are fatigued (40 % increase in energetic cost for exposed copepods swimming in calm water) or energetically impaired, especially in energetic environments such as estuaries [73].

We note that the large increase in jump frequency in turbulence observed in our measurements even over prolonged periods (1 h) is consistent with previous results obtained in the same species after a much shorter acclimation period of 5 min [27]. This sustained response to turbulence, combined with a moderate jump amplitude, tends to indicate a behavioral modulation rather than an escape response to localized hydrodynamic disturbances triggered by turbulence. Indeed, escape reactions in calanoid copepods are characterized by velocities larger than those observed in the present study [61].
